# Navigating power, participation, and priorities: critical reflections for Global Financing Facility new strategy

**DOI:** 10.1080/16549716.2025.2534250

**Published:** 2025-09-09

**Authors:** Rajat Khosla, Flavia Bustreo, Agnes Soucat, Joy Phumaphi

**Affiliations:** a Partnership for Maternal, Newborn and Child Health, World Health Organization, Geneva, Switzerland; b Fondation Botnar, Basel, Switzerland; c Agence Française de Développement, Paris, France; d African Leaders Malaria Alliance, New York, USA

**Keywords:** Global Financing Facility for Women, Children and Adolescents: Examining National Priorities, Processes and Investments, Global Financing Facility, women’s, children’s and adolescents’ health, continuum of care, equity, accountability

## Abstract

This commentary examines the learnings from different countries included in the Special Series: *Global Financing Facility for Women, Children and Adolescents: Examining National Priorities, Processes and Investments*. Studies focused on the initial phase of the GFF and highlighted key themes, including power asymmetries, stakeholder engagement, the alignment of funding to health needs, and the treatment of community health and quality of care within GFF-supported programs. This commentary reflects on policy processes and health financing dynamics emerging from the papers in the Special Series and examines what it means for the new strategy in development by the GFF. Ultimately, the GFF remains a promising model for advancing reproductive, maternal, newborn, and child health and nutrition. Realizing its transformative potential, especially with the present context, will require rebalancing technical rigor with political inclusion, and aligning performance metrics with equity-centered accountability.

## Paper Context

**Main findings:**

Realizing Global Financing Facility transformative potential, especially in the present context, requires rebalancing technical rigor with political inclusion and performance metrics with equity-centered accountability.

**Added knowledge:**

The commentary reflects on policy processes and health financing dynamics emerging from the papers in the Special Series: *Global Financing Facility for Women, Children and Adolescents: Examining National Priorities, Processes and Investments* and examines what it means for the next Global Financing Facility strategy in development.

**Global health impact for policy and action:**

Global Financing Facility remains a promising model for advancing the health of women, children, and adolescents and can play a critical role by leveraging financing models to catalyze sustainable, country-led investments in health systems by strengthening mechanisms and processes as highlighted in this Series.

## Background

The Global Financing Facility (GFF), launched by the World Bank in 2015, was seen as a landmark initiative to reduce the financing gap in reproductive, maternal, newborn, child, and adolescent health and nutrition (RMNCAH-N) through blended financing and strategic prioritization [1]. The ‘GFF model’ sought to leverage the World Bank’s International Development Association (IDA) resources and enable integration of RMNCAH-N financing into national health systems. This approach aimed to improve on earlier donor-driven mechanisms, by linking specific RMNCAH-N grant financing to World Bank IDA financing. New research published in this journal’s Special Series: *Global Financing Facility for Women, Children and Adolescents: Examining National Priorities, Processes and Investments*, reveals a more complex reality of GFF in action [2]. It offers important lessons for strengthening GFF model moving forward, aligning with the Paris, Accra, Busan and Lusaka Agenda’s call for country leadership, sustainable financing, and inclusive processes [3].

This commentary draws on the learning from this Series and reflects on what it means for the new GFF Strategy under development [4]. The studies focused on the initial phase of the GFF (2015–2022) and spotlight key themes that need to be considered for the new strategy, including power asymmetries, stakeholder engagement, funding alignment, and attention to community health and quality of care.

## Country ownership, inclusion and power relations

The country studies from Uganda, Tanzania, Mozambique and Burkina Faso highlight that while processes were framed as ‘country-led,’ the actual implementation was more complex with tension between the commitment to ‘country ownership’ and the realities of inclusion in practice [5–8]. Additionally, these studies found that the degree of consultation and transparency often varied considerably between the GFF country project documents: the Investment Case (IC) and GFF-linked World Bank Project Documents.

One key learning is that the GFF needs to align the pace of policy development with meaningful inclusion of stakeholders. The voices of country stakeholders are essential to guarantee accountability for GFF project, especially during the agenda-setting phase. This requires systemic investment in country platforms, including budget watchdogs to ensure citizens’ participation in the policy implementation. For example, the Uganda Ministry of Health rapidly developed the GFF planning and project documents within months of joining the GFF in 2015, leveraging the existing ‘A Promise Renewed’ strategy [5]. However, the speed at which this policy development process occurred limited engagement of civil society organizations, youth, faith leaders, and private sector actors. Likewise, in Tanzania’s first phase, stakeholder engagement in developing the first development phase of the project was also limited, and participation was often a ‘claimed’ rather than facilitated or meaningful engagement [6]. However, things shifted in the second phase of the GFF project development process. In Burkina Faso, despite strong political will demonstrated by the co-hosting of the 2018 GFF replenishment event, overlapping policies and fragmented donor coordination weakened the project anchoring in national systems [8].

A second key learning for the GFF is that power hierarchies also shape policy making. Using Gaventa’s Power Cube, the Tanzania study reveals visible power (e.g. funding conditionalities), hidden power (limited participatory invitations), and invisible power (neglect of certain priorities like disability or adolescent health) [6]. These imbalances are mirrored in the other countries whereby the policy making space for the World Bank project seems less transparent and less reflective of voices of country stakeholders. In Mozambique, the Primary Health Care Strengthening Program relied on disbursement-linked indicators (DLIs), which introduced technocratic complexity and potentially excluding local voices [7]. Similarly, stakeholders in Uganda and Burkina Faso also misinterpreted the ‘GFF model,’ expecting direct grants rather than catalytic funding through World Bank systems [5,8]. The disconnect between technical and political processes, combined with skewed power relations, can lead to scenarios where political capital influences financial flows even when ‘evidence-based’ mechanisms are deployed.

A key lesson for the new GFF strategy process is to strengthen inclusive, transparent, and equitable policy making the GFF country project development. In order to do this, the GFF needs to address the power imbalances and ensure meaningful participation of in-country stakeholders.

## Alignment with national priorities

Inconsistent alignment between the GFF country projects (analyzed through – the ICs and GFF-linked World Bank project documents) and national health priorities emerged as an overall finding from the four thematic content analyses [9–12]. While IC documents often reflect ambitious and comprehensive RMNCAH-N strategies, World Bank projects are narrower and focus on system-level financing or infrastructure with fewer programmatic details. For example, in six francophone West African countries, the IC documents emphasized community health and community health workers, yet these priorities were not meaningfully reflected in the GFF-linked project documents [10]. Priorities for community health in ICs were not reflected in project documents in Guinea and Côte d’Ivoire.

A key lesson is that the new GFF strategy must prioritize co-creation with local stakeholders, ensuring that investment processes reflect country-defined priorities and the commitments, as outlined in the Paris, Accra, Busan and Lusaka Agenda [3]. This includes moving beyond tokenism to embed equity and community health within financing plans; to reflect the spirit – not just the language – of the Lusaka Agenda.

## Continuum of care and of service delivery

The Series shows that some commitment to the continuum of care appears in planned projects; however, obvious gaps remain in addressing key drivers of morbidity and mortality [9,11,12].

The RMNCAH-N continuum of care starts with the health of the mother before she conceives and during pregnancy. Adolescents – who account for a large proportion of pregnancies in low-incoming countries – is the first building block and key to a healthy pregnancy and birth outcome. However, a study in this series finds adolescents’ health services inadequately addressed in several of the 16 GFF-supported countries [11]. Not only is there insufficient attention to access to family planning but there are also glaring gaps in nutrition, mental health, as well as community youth education to address substance abuse and HIV infection. These omissions pose a real danger to the health, wellbeing and quality of life of adolescents as well as to the maternal and child health.

Another study in this Series shows that critical omissions also continued into pregnancy and childbirth [9]. Stillbirths remain astonishingly high in GFF-supported countries, exacerbating mental ill health and compromising the well-being of the mother [13]. Yet, interventions specific to preventing stillbirth or underweight newborns are not explicitly mentioned despite the inclusion of antenatal care and delivery services [9]. The GFF country documents also have little emphasis on newborn and postnatal care leading to poor breastfeeding, malnutrition, poor cognitive development and stunting. Mothers have little or no support, and continue to suffer poor nutrition, and mental ill health.

A key lesson for the new GFF strategy must be to ensure all aspects of the continuum receive adequate attention, including comprehensive early childhood development and support for mothers – from pregnancy through the child’s first 5 years – to ensure healthy, productive bonds and lifelong potential.

Universal coverage with quality RMNCAH-N services at community and facility levels is the backbone of a healthy society and forms a virtuous circle that must not be broken or compromised. A well trained and supported cadre of community health workers in every community is essential; alongside functioning well-equipped, well-staffed, and well-maintained primary healthcare facilities. Moving forward, the GFF must make this approach the focus of their engagement with countries and communities.

## Centering on quality of care

Advancing quality in maternal and newborn health is one of the key commitments of GFF; however, a study in this Series finds that ‘quality’ is reported to often mentioned vaguely without definitions or specific strategies [12]. Of the 11 GFF-supported countries assessed, only Ethiopia presented a well-defined quality framework.

Where included, quality indicators focus on ‘provision of care’ (e.g. emergency obstetric care, referral systems), while overlooking ‘experience of care’ (e.g. respectful maternity practices and family-centered care). Stillbirths, a crucial indicator of maternal and newborn health quality, are rarely mentioned and unintegrated as quality measures, except in Tanzania [12]. World Bank project documents from Liberia and Uganda also offer examples linking quality goals to indicators to funding. Yet these were exceptions. Most project documents had few quality indicators and little targeted investment in improving maternal and newborn health care quality.

A key lesson for the new GFF Strategy is to define, standardize, and integrate quality of care indicators across documents, otherwise it undermines the GFF’s objective to transition from coverage to effective coverage.

## Missing in emphasis: adolescent health

As noted above, adolescent sexual and reproductive health (ASRH) is a cornerstone to advancing RMNCAH-N. The thematic content analysis focused on adolescent health shows that some ICs include ASRH and that project documents have less emphasis on it, especially in budgeting and indicator [11]. Countries with high adolescent birth rates – such as Burkina Faso, Malawi, and Senegal – provided relatively strong emphasis, but fragile or conflict-affected states had limited, if any, reference to ASRH and mental health.

Links between education, child marriage, and reproductive outcomes are acknowledged, but few documents propose school-based interventions or addressed gender inequalities. Meaningful adolescent and young people engagement in planning processes is nearly absent.

The trajectory from ‘mindset’ (policy) to ‘measures’ (indicators) to ‘money’ (budgets) shows a steep decline in attention – suggesting a missing emphasis on ASRH. The new GFF strategy must correct is missing emphasis. Lessons from the Adolescent Health Learning, Action and Benchmarking (ADLAB) collaboration with World Bank Development Economic Research Group will be important for the GFF, though the initiative still needs operational integration [14].

## Financing and resource mobilization: time for innovation

The Series shows that 30 World Bank projects linked to GFF totaled $14.5 billion from 2015 to 2022, with only 4% contribution from the GFF Trust Fund [15]. This finding demonstrates the extraordinary leveraging effect of World Bank IDA resources and other donor funding to RMNCAH-N including through concessional lending. Yet, the effect on catalyzing domestic health financing has been limited. In fact, the GFF and other global health initiatives, such as GAVI, or the Global Fund to Fight AIDS, Tuberculosis, and Malaria, may have contributed to the substitution or ‘crowding out’ of domestic resources [16]. This raises key questions as to whether the IC planning tool, often perceived as an external tool, is the most appropriate approach for domestic resource mobilization. This concern is particularly relevant in the current context of rapidly declining aid budgets.

The new GFF Strategy may want to rather focus on developing multi-annual budget programs, using country Mid-Term Expenditures frameworks (MTEFs) rather than the donor driven IC tool. An additional question to the GFF in the development of their new strategy is how to focus on poorest countries with weakest RMNCAH-N outcomes, whilst still providing incentives to middle-income countries to increase domestic financing.

The GFF has the potential to catalyze sustainable, country-led investments in health systems by mobilizing and aligning diverse financing streams through measures, such as:

- **Adjusting eligibility criteria and overall funding envelop** to be able to support all the eligible countries.
- **Enabling blended financing and co-financing arrangements** that strategically combine domestic resources, and concessional financing beyond the World Bank’s IDA and International Bank for Reconstruction and Development windows (notably through Regional Multilateral Development Banks) but also leverage funding and capacity of national Public Development Banks through extended partnerships including credit lines to optimize the efficiency and scale of health financing.
- **Facilitating private and philanthropic engagement** through mechanisms like outcome-based financing, guarantees, or pooled investment platforms aimed at social returns. These tools can complement public financing, particularly in underfunded programmatic areas.
- **Strengthening national capacity for public financial management and domestic resource mobilization**, assisting countries to develop MTEFs that include key RMNCAH-N programmes and activities and gradually increasing health spending. The aim is to align with the Lusaka Agenda’s emphasis on sovereignty and sustainability [3]. Countries should particularly consider the use of health taxes as an instrument to promote health and raise public revenues [17].
- **Supporting national platforms** that bring together citizens groups, particularly women’s groups, civil society, private sector, bilateral donors, and multilaterals, to harmonize investments with nationally defined priorities, thereby enhancing accountability and reducing fragmentation.

## Lessons on governance and sustainability

Finally, several cross-cutting governance issues emerge through the studies in this Series:

- **Institutional anchoring**: planning and budgeting processes have to have strong institutional anchoring to ensure continuity and integration into national plans.
- **Leadership and capacity**: GFF success depends on strong country leadership, technical capacity and minimizing staff turnover.
- **Accountability mechanisms**: meaningful civil society engagement in health budgeting at country level is critical to ensuring feedback loops, community accountability mechanisms and ultimately democratic governance of GFF linked WB projects.
- **Power asymmetries**: addressing power asymmetries, such as those between donors and recipients, remains key in shaping priorities, indicators, and financing terms based on local realities.

## Conclusion and recommendations

The studies in this Special Series show mixed results in achieving GFF’s ambition to transform RMNCAH-N financing through ‘country-led’ partnerships. Much has been achieved over the past decade, and progress is evident in shaping national strategies and introducing performance-based financing. However, deficiencies in stakeholder inclusion, prioritization of equity, quality of care, and misalignment between planning documents and actual implementation can undermine progress. The donor driven IC tool should particularly evolve into a more country grounded planning and budgeting model based on country budgets and MTEFs.

As the next 2026–2030 GFF Strategy is developed and implemented, key lessons from this Series should be applied:

1. **Clarify and communicate the GFF Model**: Ensure national stakeholders have a clear, early understanding of the GFF’s blended financing structure and governance implications.
2. **Expand participatory governance**: Establish mechanisms for structured engagement of civil society, youth, private sector, and affected communities – not just in consultation but in decision-making.
3. **Align quality and equity goals**: Introduce standardized quality indicators, including stillbirths and respectful care, and integrate these in planning and project documents
4. **Track financing flows transparently**: Improve budget visibility, especially for community health and adolescent programs, to ensure alignment with burden and need.
5. **Adopt flexible, contextual approaches**: Adapt models of engagement, funding, and implementation with realistic timelines for fragile and conflict-affected states.

Ultimately, the GFF remains a promising model for advancing RMNCAH-N. Realizing its transformative potential, especially with the present context, will require rebalancing technical rigor with political inclusion, and aligning performance metrics with equity-centered accountability.
